# N,N- and N,O-Bidentate-Chelation-Assisted Alkenyl C–H Functionalization

**DOI:** 10.3390/molecules30081669

**Published:** 2025-04-08

**Authors:** Yawei Zhang, Chengxing Peng, Xiaoli Li, Xiuying Liu, Liyuan Ding, Guofu Zhong, Jian Zhang

**Affiliations:** 1 School of Chemistry and Chemical Engineering, Qufu Normal University, Qufu 273165, China; 15539861693@163.com; 2 Key Laboratory of Organosilicon Chemistry and Material Technology, Ministry of Education, Zhejiang Key Laboratory of Organosilicon Material Technology, College of Material, Chemistry and Chemical Engineering, Hangzhou Normal University, Hangzhou 311121, China; 3 Department of Chemistry, Eastern Institute of Technology, Ningbo 315200, China; 4 Department of Stomatology, The Affiliated Hospital of Hangzhou Normal University, Hangzhou Normal University, Hangzhou 310015, China

**Keywords:** C–H activation, bidentate chelation assistance, directing group, alkene

## Abstract

Chelation-assisted olefinic C–H functionalization has been demonstrated to be a powerful method of constructing multi-substituted alkenes from simpler ones. This strategy produces complex alkenes in a regio- and stereoselective manner, followed by C–H endo- and exo-cyclometallation. Among the various directing groups developed, N,N- and N,O-bidentate directing groups are the most widely used to selectively promote C–H functionalization due to their fine, tunable, and reversible coordination with the metal center. In this review, we discuss various N,N- and N,O-bidentate directing group-assisted olefinic C–H bond functionalization reactions, including alkenylation, alkylation, arylation, thiolation, silylation, halogenation, and cyclization.

## 1. Introduction

Chelation-assisted C–H functionalization has been demonstrated to be a powerful method for the construction of complex structures [1,2,3,4,5,6,7,8,9,10,11]. These methods exhibited excellent regio- and stereoselectivity by distinguishing the C–H bonds of the directing groups (DGs) with different distances and geometries. These reactions are proceeded by C–H activation to form five- and six-membered metallocycles, which exhibit excellent regio- and stereo selectivity. It is well known that the DGs usually play specific roles, such as coordinating to transition metal to bring it next to the target C–H bond and improving the reaction efficiency by increasing the catalyst concentration, to promote C–H functionalization. Many efforts have been made to achieve chelation-assisted aromatic [12,13,14] and aliphatic C–H functionalization [15,16]. In contrast, alkenyl C–H functionalization has attracted much less attention presumably due to issues such as the easier decomposition and higher reactivity of olefin substrates than arene and alkane substrates [17,18,19,20,21,22,23]. For example, the easier coordination of the alkene to the transition metal competes with olefinic C–H activation. The absence of the aromatic delocalization of the alkenyl C–H cyclometallation also makes them more challenging.

**Scheme 1:**
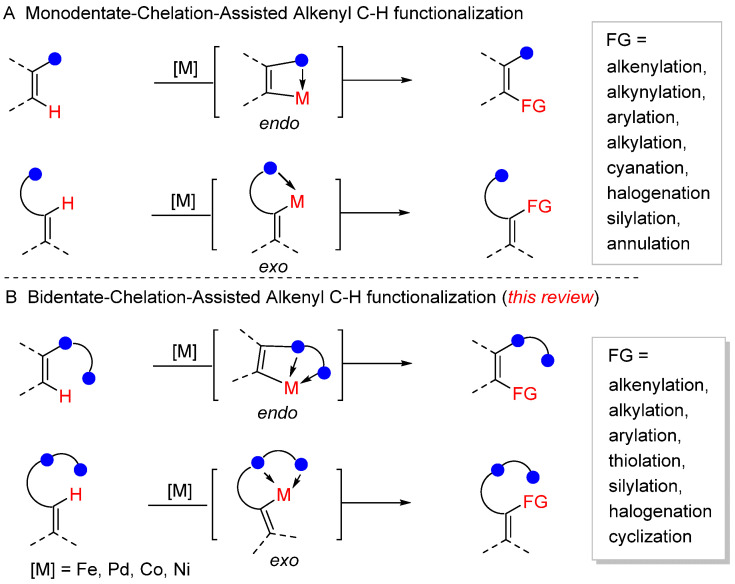
(**A**) Monodentate-chelation-assisted alkenyl C–H functionalization. (**B**) Bidentate-chelation-assisted alkenyl C–H functionalization.

Although chelation-assisted olefinic C–H activation is difficult, remarkable progress in the construction C−C and C−X bonds has been made by the adjustment of reaction substrates and catalytic conditions. There are two types of alkenyl C–H functionalization, which are determined according to the C–H cyclometallation event as well as the formation of endo- and exo-metallocycles [17,18,19,20,21,22,23]. As directing groups play key roles in alkenyl C–H functionalization, many mono-bidentate-chelation DGs such as amide, carboxylic acid, ester, and hydroxyl have been developed to ensure excellent selectivity in olefinic C–H alkenylation, alkynylation, arylation, alkylation, cyanation, halogenation, silylation, and annulation. While monodentate functionality directed C–H bond functionalization has been extensively explored, drawbacks have been observed such as the occurrence of uncontrolled reactions or the extremely low reactivity of the olefinic substrate due to the weak coordination ability of a monodentate directing group to a transition metal (Scheme 1A). In this regard, it is highly desirable to introduce bidentate directing groups to tune the coordination ability to the metal center to promote reactivity.

The first example is the N,S-bidentate directing group, which enables ortho-C–H iodination via palladium catalysis [24]. Subsequently, Daugulis et al. firstly developed N,N-bidentate directing groups such as quinolinamide and picolinamide to achieve C–H arylation [25], which were also utilized for aromatic and alkyl C–H alkylation, allylation, alkenylation, alkynylation, and cyclization [26]. Although these bidentate directing groups exhibit many advantages, the installation and removal of DGs are tedious and decrease the synthesis efficiency. In this regard, the use of a transient bidentate directing group improves the step economy in olefinic C–H bond functionalization [27,28,29].

In this review, we provide an in-depth summary of alkenyl C–H bond functionalization using bidentate directing groups, and the transformations are classified based on the N,N- and N,O-bidentate directing groups [30,31,32,33,34,35,36,37,38,39,40,41,42,43,44,45,46,47,48,49,50,51,52,53,54,55,56,57,58,59,60,61,62]. Moreover, the reaction pattern is further subcategorized as alkenylation, alkylation, arylation, thiolation, silylation, halogenation, and cyclization (Scheme 1B).

## 2. Bidentate-Chelation-Assisted Alkenyl C–H Functionalization

While bidentate-chelation-assisted directing groups have been demonstrated to be widely applicable for aromatic and alkyl C–H functionalization, they have also been successful in enabling alkenyl C–H functionalization. N,N-bidentate-chelation-assisted directing groups such as 8-quinolylamide and picolinamide group have been widely used in Fe-, Pd-, Ni-, or Co-catalyzed olefinic C–H functionalization such as alkenylation, alkylation, arylation, thiolation, silylation, and halogenation.

### 2.1. N,N-Bidentate-Chelation-Assisted Alkenyl C–H Functionalization

#### Alkenyl C–H Alkylation

**Scheme 2:**
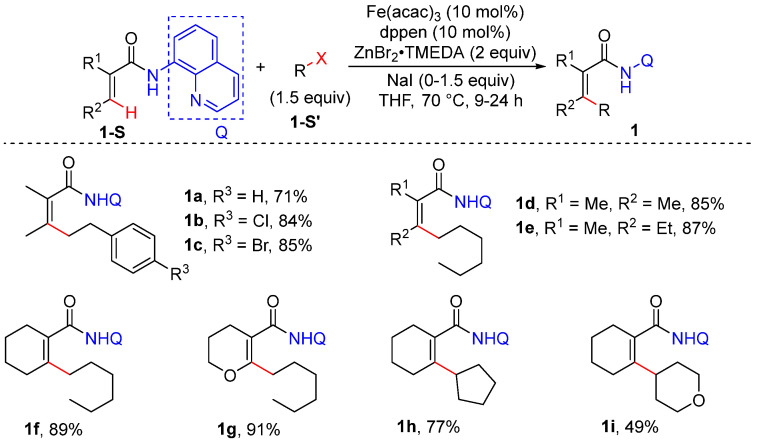
Fe–catalyzed alkenyl C–H alkylation using alkyl halides.

In 2014, Ilies et al. developed a method for the iron-catalyzed alkenyl and aromatic C–H alkylation of acrylamides bearing an 8-quinolylamide group, using primary and secondary alkyl tosylates, mesylate, and halides as the alkylation reagents [30]. This reaction proceeds smoothly using Fe(acac)_3_ and cis-1,2-bis(diphenyl phosphino) ethylene (dppen) as the catalyst and ArZnBr as the base, leading to excellent regio- and stereoselectivity. Other directing group such as 2-methyl-8-aminoquinoline, N-phenylamide, and pyridine all resulted in low reactivity. This transformation is sensitive to the ligand. Diphosphine possessing a π bridge such as dppen efficiently promotes the reaction; however, 1,2-ethylene diphosphine (dppe), lacking the π bridge, and monophosphine are ineffective (Scheme 2).

**Scheme 3:**
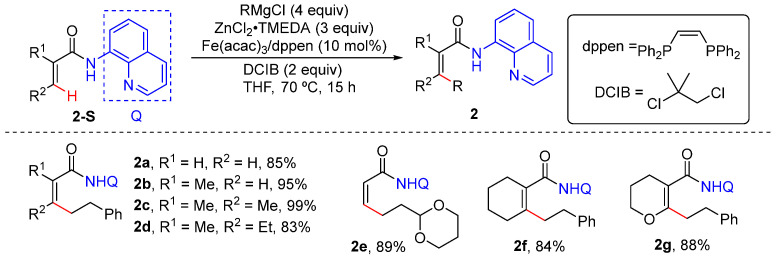
Fe-catalyzed alkenyl C–H alkylation using alkyl Grignard reagent.

In 2015, Ilies and Nakamura reported the iron-catalyzed alkenyl C–H alkylation of unsubstituted and disubstituted acrylamides bearing an 8-quinolylamide group, using primary and secondary alkylzinc halides generated from Grignard reagent [31]. This reaction introduces Fe(acac)_3_ and cis-1,2-bis(diphenyl phosphino) ethylene (dppen) as the catalyst and dichloroalkane as the oxidant. Among the 4 equiv. of Grignard reagent required, 3 equiv. is used to generate an organozinc halide, and the remaining 1 equiv. is introduced to deprotonate the amide to facilitate coordination. Among the excess amount of organozinc halide required, 1 equiv. acts as the alkylating reagent and 1 equiv. is used as a base to remove the β-hydrogen of acrylamide (Scheme 3).

**Scheme 4:**
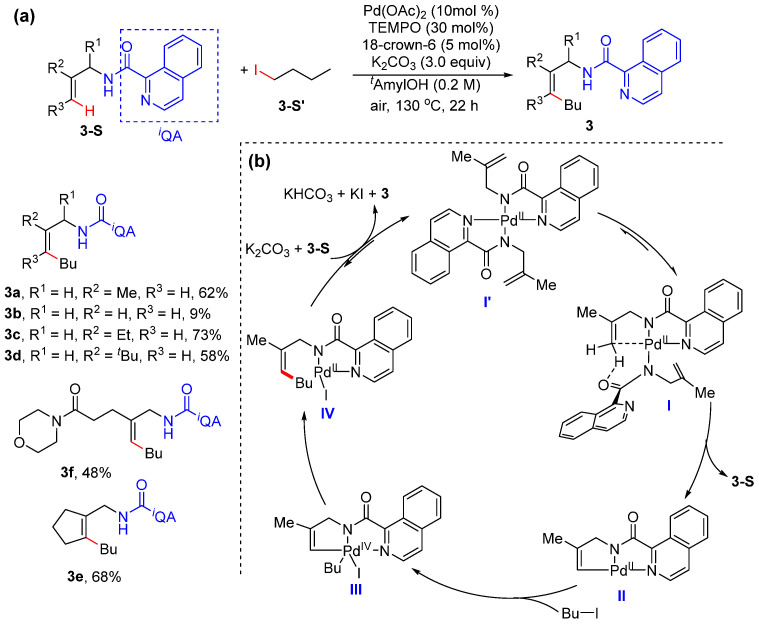
(**a**) Pd-catalyzed alkenyl C–H alkylation using alkyl iodides. (**b**) Proposed mechanism.

In 2019, Loh and Xu reported the palladium-catalyzed olefinic C–H alkylation of allylamines using primary and secondary alkyl iodides with the assistance of a directing group, isoquinoline-1-carboxamide (IQA) [32]. The frequently used promoters such as (BnO)_2_PO_2_H and PivOH do not work efficiently in the reaction. Interestingly, 2,2,6,6-tetramethylpiperidine-1-oxyl (TEMPO) and 18-crown-6(18-Cr-6) significantly improve the reaction efficiency (Scheme 4a). The kinetic isotope effect was determined to be 3.3, showing olefinic C–H activation to be the rate-determining step. The initial reaction rate is determined depending on the concentrations of the catalyst and substrates, suggesting that the palladium catalyst is involved in olefinic C–H activation, and the substrates might not participate in the rate-determining step. A plausible catalytic cycle may be initiated by the generation of complex **I’** from Pd(OAc)_2_ and allylamide, which undergo isomerization to give rise to intermediate **I**. The following concerted base-assisted metalation−deprotonation occurs to produce intermediate **II**, which reacts with iodoalkane via oxidative addition to generate a Pd(IV) species **III**. The reductive elimination of **III** occurs to produce **3**, and the catalyst is regenerated in the presence of K_2_CO_3_ and another allylamide (Scheme 4b).

**Scheme 5:**
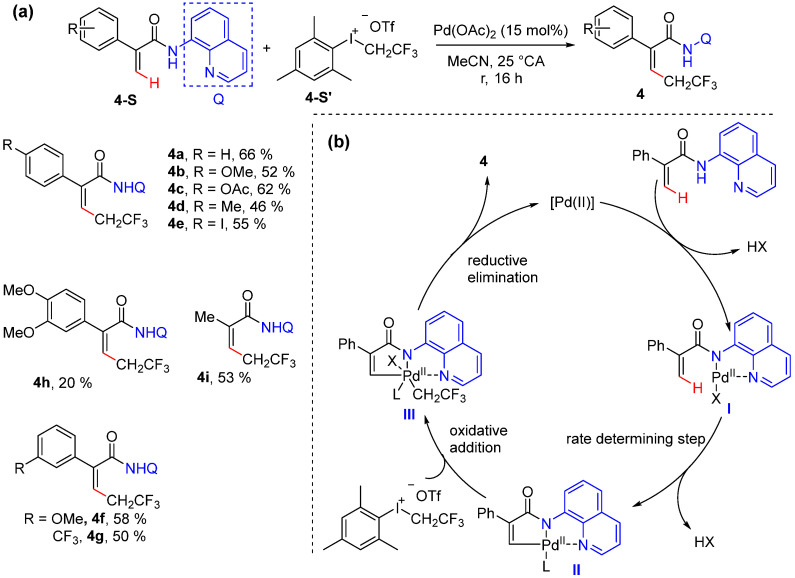
(**a**) Pd-catalyzed alkenyl C–H alkylation using hypervalent iodine. (**b**) Proposed mechanism.

In 2021, Besset’s group reported the Pd-catalyzed 2,2,2-trifluoroethylation of acrylamides bearing an 8-quinolylamide group, which was achieved using a fluorinated hypervalent iodine as a coupling partner [33]. This reaction is simply performed at room temperature without any additive using 15% Pd(OAc)_2_ in MeCN. Mechanistic experiments ruled out a free radical pathway, as the addition of 3,5-di-tert-4-butylhydroxytoluene (BHT) did not influence the reaction efficiency (Scheme 5a). H/D scrambling experiments and KIE experiments suggested that alkenyl C–H activation is irreversible and probably the rate-determining step. The reaction is proposed to proceed via the coordination of the palladium catalyst to the directing group and the formation of the palladacyle intermediate presumably via a C–H concerted metalation–deprotonation (CMD) process. The subsequent oxidative addition with hypervalent iodine followed by reductive elimination give rise to the desired product and regenerate the catalyst (Scheme 5b).

**Scheme 6:**
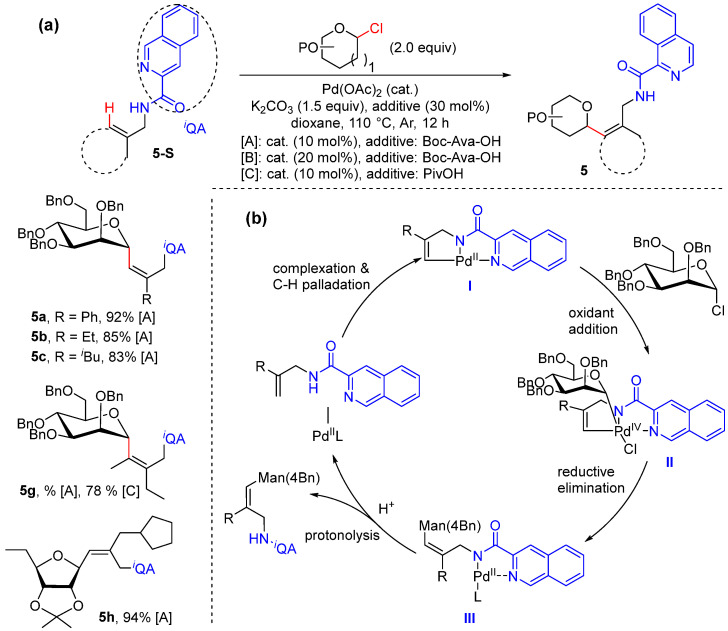
(**a**) Pd-catalyzed alkenyl C–H glycosylation using glycosyl chlorides. (**b**) Proposed mechanism.

C-vinyl glycosides represent a class of carbohydrates of unique importance. In 2021, He and Chen reported the Pd-catalyzed stereoselective synthesis of C-vinyl glycosides via alkenyl C–H glycosylation using glycosyl chloride donors with chelation assistance provided by auxiliary N,N-bidentate (Scheme 6a) [34]. Both the allylamines and homoallylamine substrates can be converted with high efficiency and have excellent regio- and stereoselectivity. Deuterium-labeled experiments exhibited the C–H palladation step to be reversible and the rate-limiting step. The reaction is proposed to proceed via alkenyl C–H palladation to form the palladacycle intermediate **II**; oxidative addition with glycosyl chloride to **II** gives the Pd(IV) intermediate **III**, which then gives rise to the glycosylated product **IV** via reductive elimination (Scheme 6b).

### 2.2. Alkenyl C–H Alkenylation

**Scheme 7:**
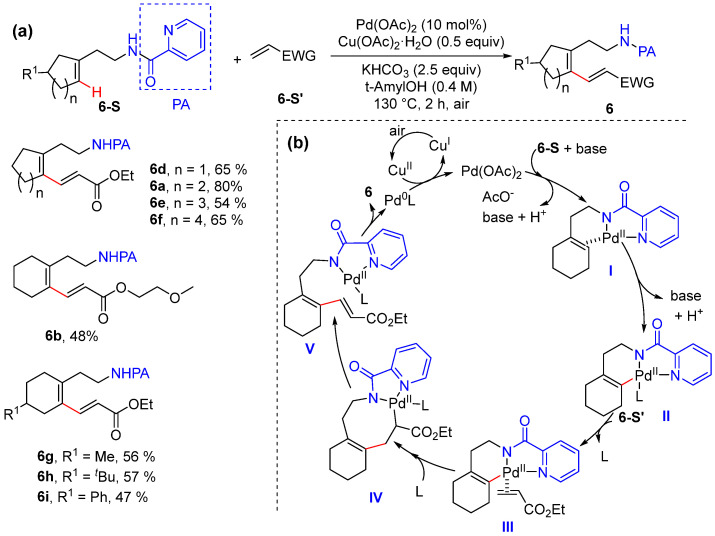
(**a**) Pd-catalyzed alkenyl C–H alkenylation of cyclic alkenes. (**b**) Proposed mechanism.

In 2020, He and Cai achieved the palladium-catalyzed alkenyl C–H alkenylation of cycloalkenes using electron-deficient alkenes as coupling partners [35]. The reaction employs Pd(OAc)_2_ as the catalyst and Cu(OAc)_2_·H_2_O as the oxidant to give 1,3-dienes with excellent site- and stereoselectivity, using picolinamide as a bidentate directing group to promote the formation of a unique six-membered alkenyl palladacycle intermediate (Scheme 7a). The reaction exhibits broad substrate scope including five-, six-, seven-, and eight-membered cyclic alkene substrates. A plausible mechanistic cycle starts with the formation of π-alkene palladium complex **I,** followed by alkenyl C–H activation to produce six-membered palladacycle **II**. The ligand exchange of the electron-deficient alkene and migratory insertion give intermediate **IV**, and syn β-H elimination and reductive elimination produce **6** and Pd(0) species, which are converted to catalytically active Pd(II) species via oxidation with Cu(OAc)_2_·H_2_O (Scheme 7b).

**Scheme 8:**
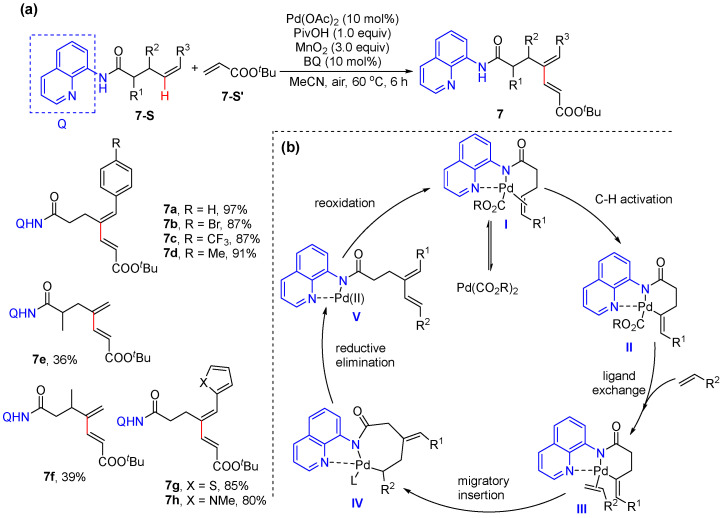
(**a**) Pd-catalyzed alkenyl C–H alkenylation of aliphatic alkenes. (**b**) Proposed mechanism.

In 2018, Liu and Engle reported the N,N-bidentate-chelation-assisted alkenyl C–H alkenylation of Z-alkenes derived from 4-pentenoic acids, allylic alcohols, and bishomoallylic amines bearing Daugulis’s 8-aminoquinoline (Scheme 8a) [36]. The reaction is performed using 10 mol% Pd(OAc)_2_ and stoichiometric MnO_2_ or catalytic Co(OAc)_2_ under O_2_. A plausible catalytic cycle was proposed to proceed via alkene coordination to metal to form π-alkene palladium complex **I**, and the following alkenyl C–H activation occurs to produce six-membered palladacycle **II**. The subsequent coordination of the electron-deficient alkene and migratory insertion afford eight-membered palladacycle intermediate **IV**. Finally, β-H elimination and reoxidation of the palladium catalyst by the oxidant give diene product **7** and regenerate the catalytically active species (Scheme 8b).

**Scheme 9:**
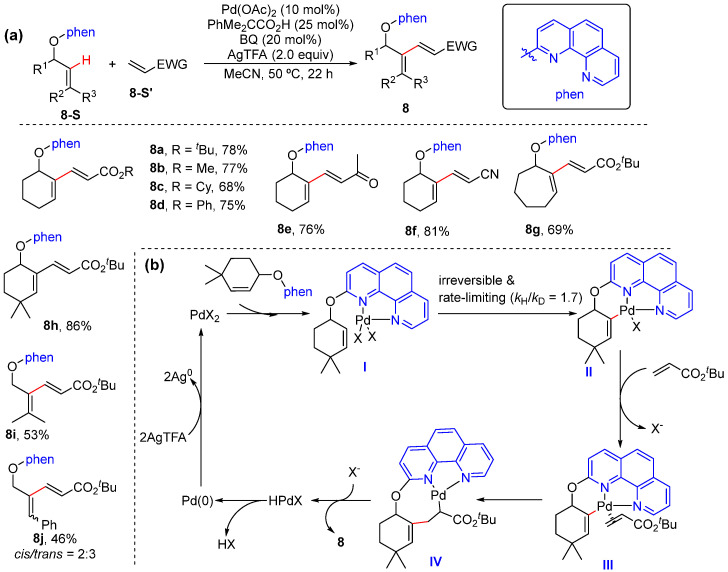
(**a**) Pd-catalyzed alkenyl C–H alkenylation of allyl ethers. (**b**) Proposed mechanism.

In 2020, Hirano and Miura achieved the Pd-catalyzed alkenyl C–H alkenylation of allylic-alcohol-derived ethers bearing phenanthroline using electron-deficient alkenes (Scheme 9a) [37]. In this transformation, the N,N-bidentate coordinating phenanthroline plays a key role to enable selective alkenyl C–H bond activation and avoid competitive allylic C−O bond activation. The Pd/phenanthroline catalytic system is efficient at alkenyl C–H alkenylation as well as alkynylation. The mechanism is proposed to proceed via the coordination of the phenanthroline moiety to the palladium center, followed by alkenyl C–H cleavage to form 6-membered palladacycle intermediate **II**. Next, ligand exchange on palladium takes place, followed by olefin insertion and β-H elimination, to afford alkenylation product **8**, with the liberation of HX and Pd(0), which undergo reoxidation to complete the catalytic cycle (Scheme 9b). Mechanistic experiments showed the olefinic C–H bond activation to be irreversible and the rate-limiting step. A concerted metalation–deprotonation mechanism was proposed to occur due to the positive effects of acid.

**Scheme 10:**
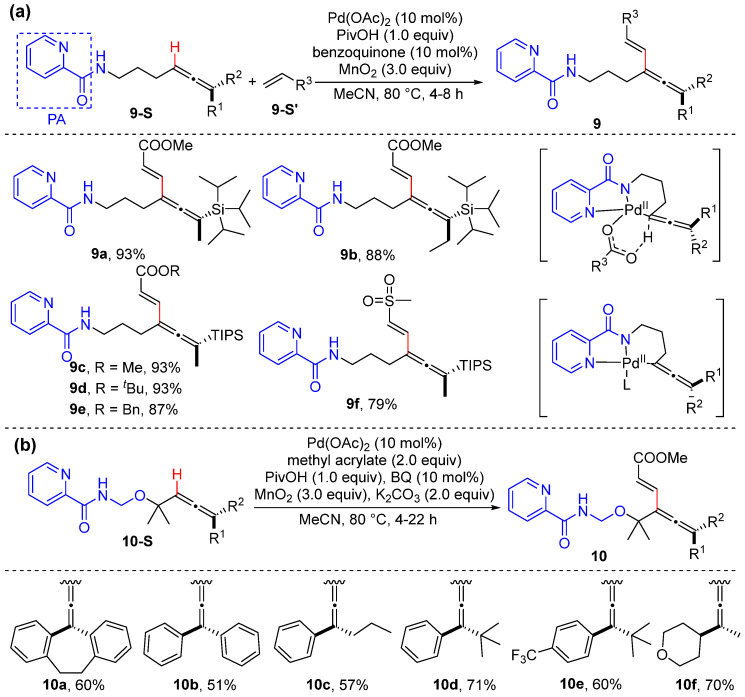
(**a**) C–H alkenylation of bishomoallenylic picolinamides. (**b**) C–H alkenylation of allenylic alcohols.

Baik and Carreira achieved the Pd-catalyzed olefinic C–H alkenylation of electronically unbiased allenes using picolinamide as the directing group [38]. This reaction proceeds via a putative six-membered allenyl-palladacycle, using electron-deficient alkenes as coupling partners. Allyl-alcohol-derived N,O-acetals bearing picolinamide are also suitable substrates for C–H functionalization. The reaction is considered to proceed through a key concerted metalation−deprotonation step, which is supported by a combination of experimental and computational approaches with allene C−C π-bond intact. Notably, the picolinamide group can be readily converted into N-Boc-protected amine with high efficiency (Scheme 10).

Aryl alkenes are widely used chemicals, and their C–H functionalization has been extensively studied. However, previous work has focused on β-C–H functionalization, and reports are rare on α-C–H functionalization via exo-cyclomatallation [22].

**Scheme 11:**
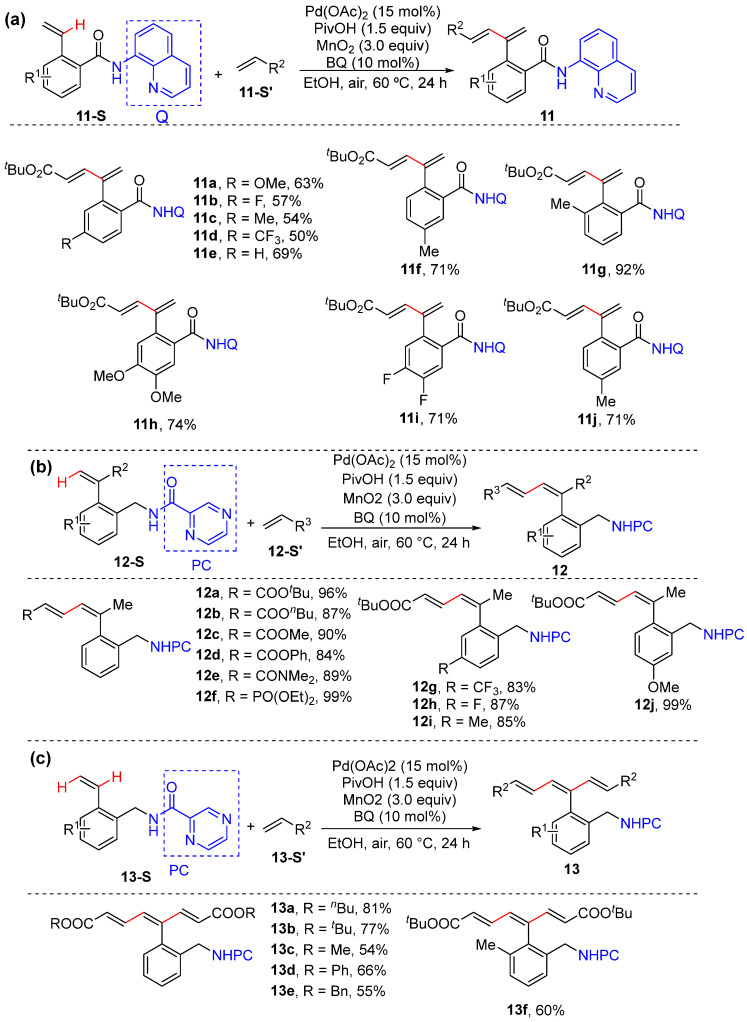
(**a**) Pd-catalyzed α-C–H alkenylation of plain styrenes. (**b**) β-C–H alkenylation of α-substituted styrenes. (**c**) α- and β-bis C–H alkenylation of plain styrenes.

In 2022, Zhong and Zhang reported the N,N-bidentate-chelation-assisted α- and/or β-C–H alkenylation of plain- and α-substituted aryl alkenes to give aryl dienes and trienes with excellent site- and stereoselectivity (Scheme 11a–c) [39]. This method employs 2-alkenyl benzylamine and benzoic acid derivatives bearing Daugulis’s 8-aminoquinoline (AQ) and 2-pyrazinamide (PC) as substrates, which is performed using a combination of a catalytic amount of Pd(OAc)_2_ and benzoquinone (BQ), a quantitative amount of MnO_2_ as the oxidant, and PivOH as the additive in ethanol. Interestingly, plain 2-vinyl benzamides bearing 8-aminoquinoline (AQ) only lead to α-C–H alkenylation (Scheme 11a); however, α,β-bis C–H alkenylation takes place smoothly to produce 1,3,5-trienes if plain 2-vinyl benzamides bearing 2-pyrazinamide (PC) are used as the substrates instead (Scheme 11c). The reactions are proposed to proceed via six-membered exo-cyclopalladation and seven-membered endo-cyclopalladation.

**Scheme 12:**
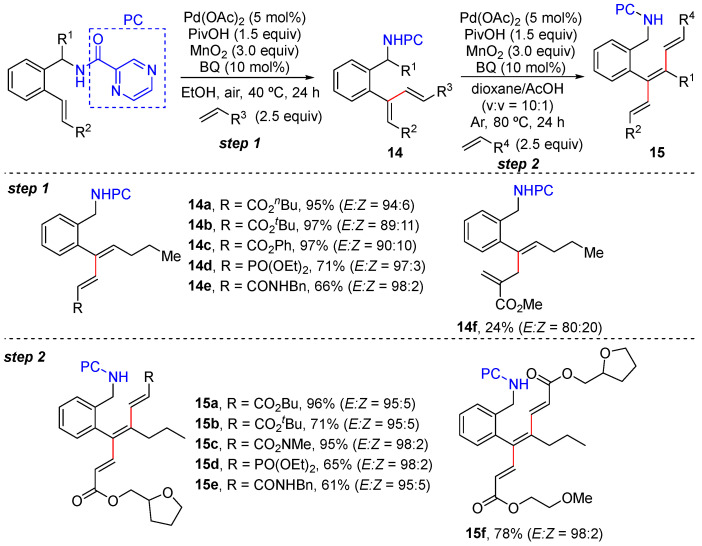
Pd-catalyzed sequential α- and β-C–H alkenylation of *trans*-styrenes.

In 2024, Zhang and Zhong reported the sequential α- and β-C–H alkenylation of trans-styrenes bearing 2-pyrazinamide as the directing group, efficiently producing 1,3-dienes and trienes with excellent regio- and stereoselectivity [40]. The reactions were performed using 5 mol% Pd(OAc)_2_, 10 mol% *p*-benzoquinone (*p*-BQ), 1.5 equivalents of PivOH, and 3.0 equivalents of MnO_2_. α- and β-C–H functionalization proceeds via the formation of six-membered exo-palladacycle and seven-membered endo-palladacycle, with up to 97% yield and up to >99/1 E/Z selectivity, both of which are enabled via chelation assistance with pyrazinamide. The method tolerates a wide range of functional groups and is suitable for gram-scale preparation as well as the one-pot and two-step preparation of trienes. Mechanistic experiments showed that C–H exo-cyclopalladation is reversible and alkenyl C–H cleavage is the rate-determining step. Competition experiments using cis- and trans-styrenes exhibited that exo-cyclopalladation with a trans-substrate is much more difficult (Scheme 12).

**Scheme 13:**
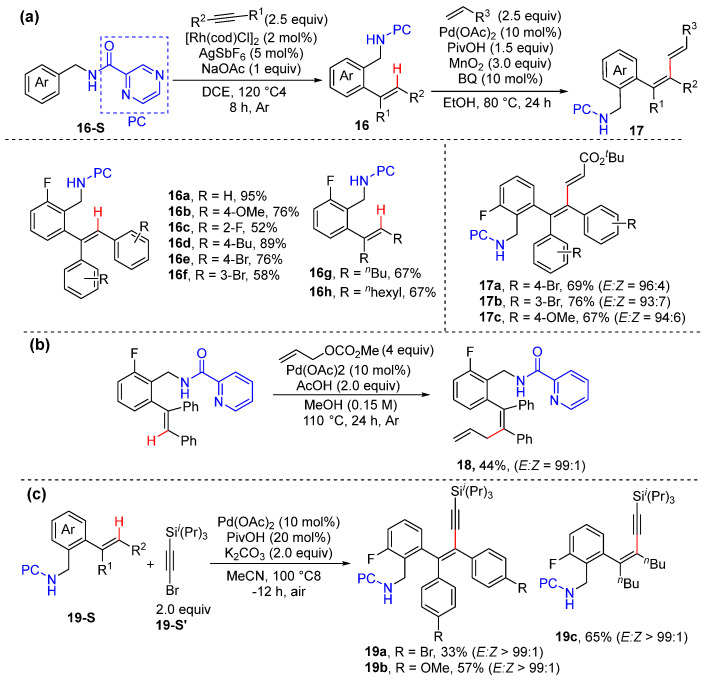
(**a**) Sequential hydroalkenylation and alkenyl C–H alkenylation. (**b**) Alkenyl C–H allylation. (**c**) Alkenyl C–H alkynylation.

Very recently, Zhong and Zhang achieved sequential aromatic C–H alkenylation via the hydroarylation of the internal alkynes and olefinic C–H functionalization, including alkenylation, alkynylation, and allylation, all enabled by the N,N-bidentate-chelation-assistance of pyrazinamide or picolinamide [41]. The first step in the protocol proceeds with five-membered rhodacycle, and the subsequent olefinic C–H functionalization proceeds through seven-membered endo-cyclopalladation, producing to complex 1,3- and 1,4-dienes and 1,3-eneynes with excellent site- and stereoselectivity. The photophysical properties of the products were also investigated, and interesting AIE activity and luminescence properties were observed (Scheme 13).

**Scheme 14:**
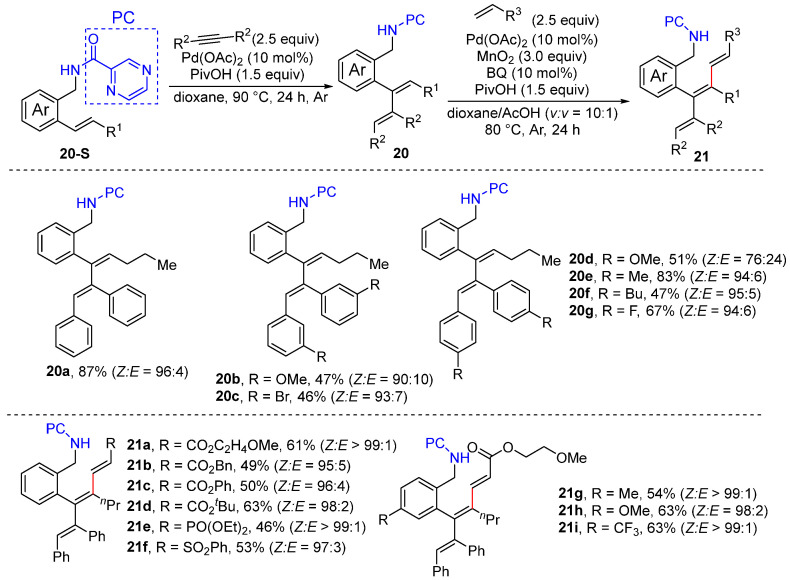
Pd-catalyzed alkenyl hyroalkenylation and C–H alkenylation.

Given our ongoing interest in olefinic C–H functionalization, we also achieved the palladium-catalyzed α C–H alkenylation of trans- and cis aryl alkenes via the hydroalkenylation of the internal alkynes, simply performed using 10 mol% Pd(OAc)_2_ and 1.5 equivalents of pivalic acid in 1,4-dioxane [42]. The reactions proceed via the formation of a six-membered exo-palladacycle to produce conjugated dienes with excellent E/Z ratio selectivity. The diene product prepared from trans-aryl alkenes is also converted to trienes via oxidative β C-H alkenylation through a seven-membered endo-palladacycle. The chemical derivatization and photophysical properties of the polyene products were examined. The photophysical properties were also investigated, showing potential applications in OLEDs and living animal imaging through further structural derivations (Scheme 14).

### 2.3. Alkenyl C–H Allylation

Although many efforts have been made to achieve mono-dentate-chelation-assisted alkenyl C–H allylation, reports are rare on N,N-bidentate-chelation-assisted C–H allylation.

**Scheme 15:**
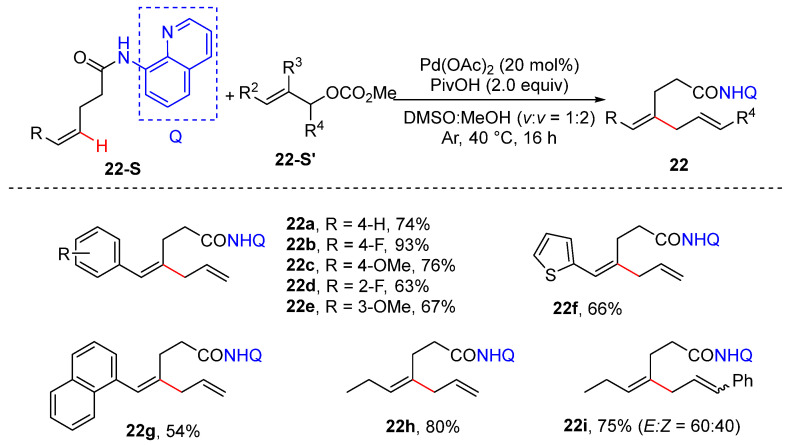
Pd-catalyzed alkenyl C–H allylation of *Z*-alkenes.

In 2019, Zhong and Zhang achieved the alkenyl C–H allylation of inactivated (Z)-alkene and allyl carbonates, which was simply performed using 20 mol% Pd(OAc)_2_ and 2 equivalents pf PivOH in mixed DMSO/MeOH (*v*/*v* 1:2) under argon [43]. Different allylation reagents were examined. While allyl alcohol, allyl iodide, and allyl phosphate showed significantly lower reactivity, the other allyl carboxylic esters led to moderate yield with undesired double-bond migration products. A kinetic isotope effect (KIE) value of 2.6 was determined via an intermolecular competition experiment, suggesting alkenyl C–H cleavage to be the rate-determining step. The 8-aminoquinolin amide group can be conveniently removed under Ohshima’s methanolysis conditions via nickel catalysis to produce ester in good yields (Scheme 15).

**Scheme 16:**
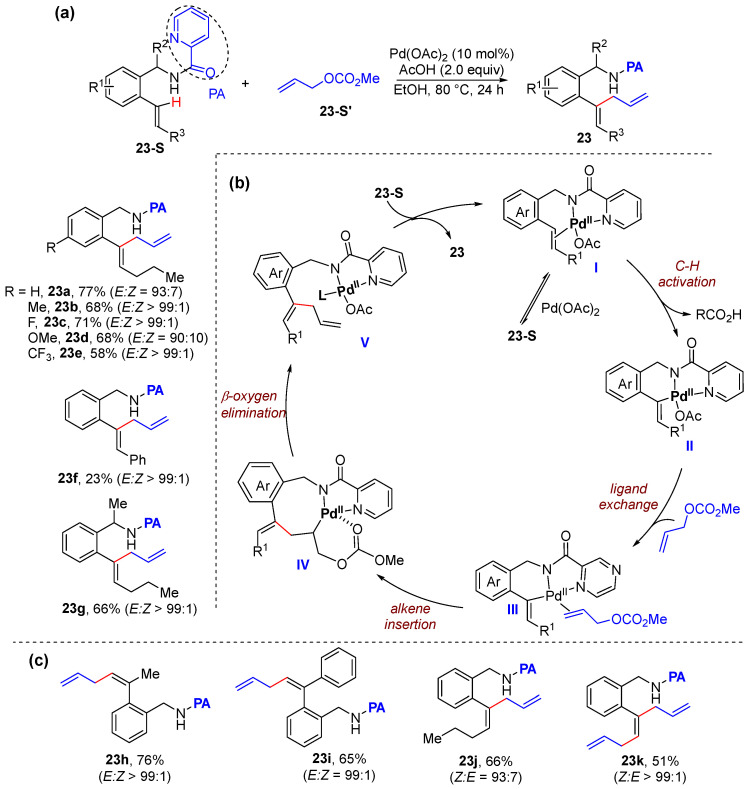
(**a**) Pd-catalyzed alkenyl α-C–H allylation of aryl alkenes. (**b**) Proposed mechanism. (**c**) β-C–H allylation of aryl alkenes.

Very recently, the same group also achieved the first α- and β-C–H allylation of aryl alkenes to prepare linear and branched 1,4-dienes using allyl carbonates as the allylation reagents [44]. The C–H allylation reaction is enabled by pyridine-2-carboxamide as the directing group, which is performed using Pd(OAc)_2_ as the catalyst an AcOH as the promoter in ethanol (Scheme 16a). This protocol can be applied to a broad substrate scope such as E- and Z-configurated aryl alkenes, plain styrene, as well as α-substituted styrenes. The mechanism includes the coordination between substrate and palladium, C–H activation to form six-membered palladacycle **II**, ligand exchange and alkene insertion with allyl carbonate, and β-oxygen elimination to produce 1,4-diene **23** (Scheme 16b). This protocol is also successfully applied to β-C–H allylation of aryl alkenes (Scheme 16c).

Both of the protocols are operationally simple and employ PivOH or AcOH as an additive to promote alkenyl C–H activation.

### 2.4. Alkenyl C–H Alkynylation

Alkenyl C–H alkynylation is a powerful method for the preparation of 1,3-eneynes with excellent E/Z ratio selectivity. There are only two examples on alkynylation using palladium or nickel catalysis.

**Scheme 17:**
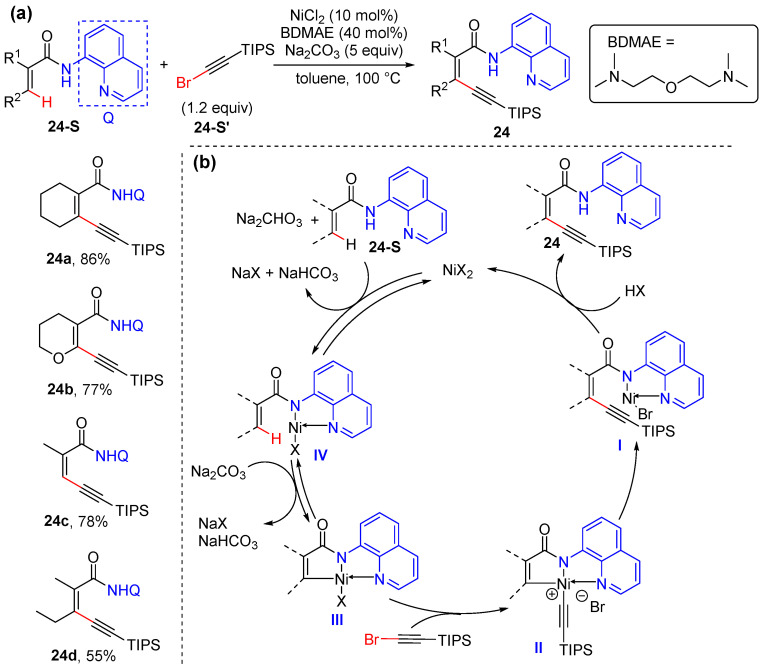
(**a**) Ni-catalyzed alkenyl C–H alkynylation of acrylamides using bromoalkyne. (**b**) Proposed mechanism.

In 2015, Li’s group developed a process for the efficient nickel-catalyzed alkenyl C–H alkynylation of acrylamides with the directing assistance of 8-aminoquinoline [45]. Acyclic and cyclic acrylamides are both tolerated, as well as mono- and disubstituted ones. A simple salt NiCl_2_ (10 mol%) and bis(2-dimethylaminoethyl) ether (BDMAE) (40 mol%) are used to catalyze the reaction, using 5 equivalents of Na_2_CO_3_ in toluene, and the catalytic conditions were also successfully applied for aromatic C–H alkynylation (Scheme 17a). The directing group is readily removed to give aldehyde or ester in high yields. The possible mechanism starts with the coordination of amide to the nickel catalyst, followed by ligand exchange and then C–H cyclometallation, to give five-membered metallocycle intermediate **II**. Subsequently, the oxidative addition of bromoalkyne produces high-valent Ni(IV) species **III**, which undergoes reductive elimination and protonation to yield 1,3-eneyne **24** and liberates the Ni(II) catalyst (Scheme 17b).

**Scheme 18:**
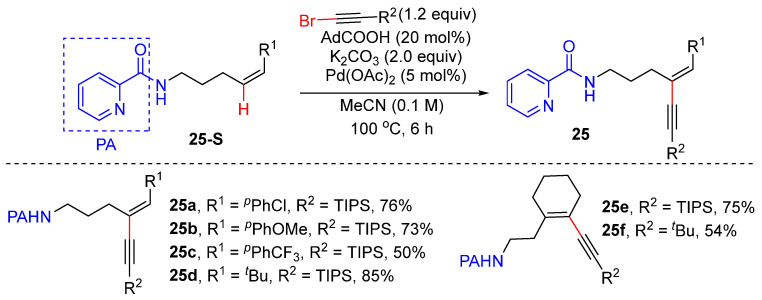
Pd-catalyzed alkenyl C–H alkynylation of aliphatic *Z*-alkenes using bromoalkyne.

In 2020, Carreira’s group achieved the Pd-catalyzed regio- and E/Z-selective alkenyl C–H alkynylation of electronically unbiased Z-alkenes with bromoalkynes to produce 1,3-eneyne **25** [46]. A picolinamide directing group was used to enable the formation of putative five and six-membered exo-palladacycle. Alkenyl C–H alkynylation was successfully achieved by using 5 mol% Pd(OAc)_2_, 2 equivalents of K_2_CO_3_, and 20 mol% pivalic acid in MeCN as the solvent. The picolinamide directing group was readily removed by treating with Zn/HCl to produce free amine (Scheme 18).

### 2.5. Alkenyl C–H Arylation

Alkenyl C–H arylation provides an efficient method for the synthesis of aryl alkenes. Most of the reported work on C–H arylation has employed palladium catalysis with the aid of silver salt [47,48,50,51,52], and there is only one example using the inexpensive transition metal, cobalt [49].

**Scheme 19:**
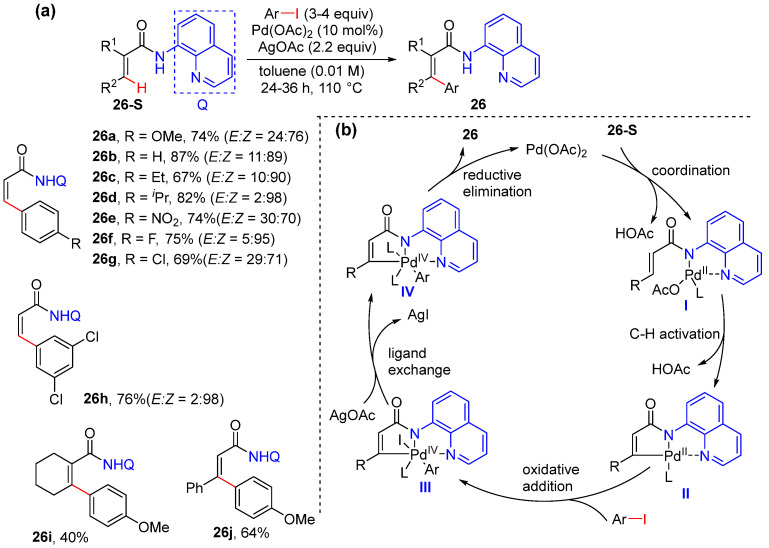
(**a**) Pd-catalyzed alkenyl C–H arylation of acrylamides achieved by Babu et al. (**b**) Proposed mechanism.

In 2015, Babu’s group reported the Pd-catalyzed alkenyl C–H arylation using Pd(OAc)_2_ as the catalyst and AgOAc as the promoter in toluene as the solvent [47]. In the presence of 4 equivalents of aryl- and heteroaryl iodides, the arylation reaction proceeded smoothly to produce a Z-selective product with moderate to excellent E/Z selectivity (Scheme 19a). The reaction was proposed to start with N,N-bidentate coordination of the N-(quinolin-8-yl)acrylamide to palladium, followed by alkenyl C–H activation, to afford five-membered palladacycle intermediate **II**. Oxidative addition of **II** with ArI delivers Pd(IV) species **III**, and the final reductive elimination of the Pd(IV) species produces the desired aryl alkene **26** (Scheme 19b).

**Scheme 20:**
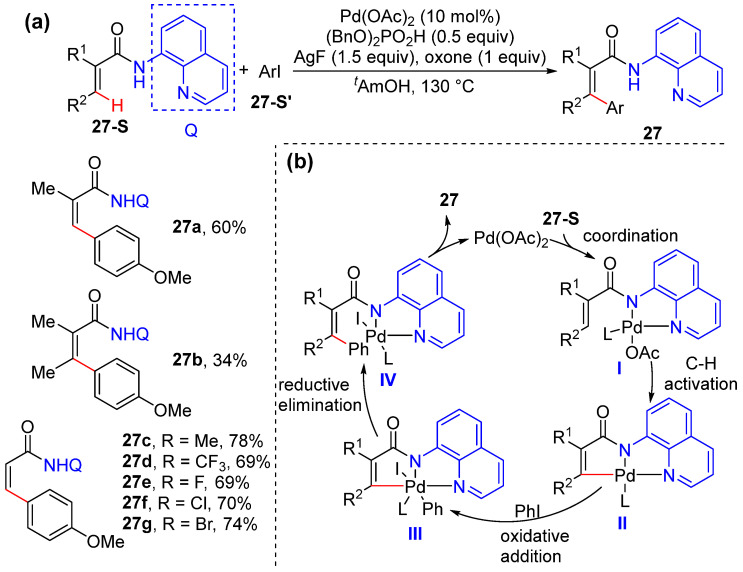
(**a**) Pd-catalyzed alkenyl C–H arylation of acrylamides according to Zhu. (**b**) Proposed mechanism.

Xue and Jiang reported a similar method for Pd-catalyzed vinylic C–H bond arylation using aryl iodides under the assistance of aminoquinoline groups [48]. This reaction was performed using 10 mol% Pd(OAc)_2_, 1.5 equivalents of AgF, 0.5 equivalents of (BnO)_2_PO_2_H, and 1.0 equivalent of oxone (Scheme 20a). The conditions were successfully applied to mono-, di-, and unsubstituted acrylamides. A Pd(II)/Pd(IV) catalytic cycle was proposed including C–H cyclometallation via directed olefinic C–H activation, the oxidative addition of aryl iodide to form Pd(IV) complexes, and reductive elimination and ligand exchange to liberate product **27**. Also, the electronic properties of aryl iodides have little effect on reactivity, which was confirmed by intermolecular competition experiments (Scheme 20b).

**Scheme 21:**
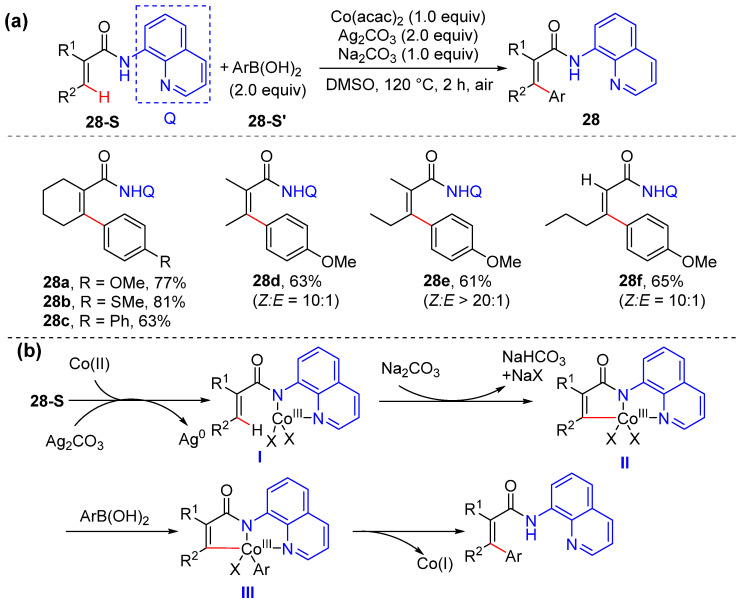
(**a**) Co-promoted alkenyl C–H arylation of acrylamides. (**b**) Proposed mechanism.

Tan and Zhu achieved the cobalt-promoted arylation of olefinic and aryl C–H bonds with aryl boronic acids by using 8-aminoquinoline as the directing group [49]. The reaction tolerates a wide range of functional groups and can be applied with diversely substituted substrates (Scheme 21a). A plausible mechanism including Co(II)–Co(III)–Co(I) starts with the oxidation of Co(II) to Co(III) by Ag_2_CO_3_ and ligand exchange to give intermediate **I**, which undergoes olefinic C–H activation to form five-membered alkenyl Co(III) species **II**. The subsequent trans-metallation of **II** with phenylboronic acid occurs to produce cobalt complex **III**, and the final reductive elimination of the Co(III)–complex **III** delivers the desired product **28**. However, the Co(II)−Co(IV)−Co(II) mechanistic cycle is also possible (Scheme 21b).

**Scheme 22:**
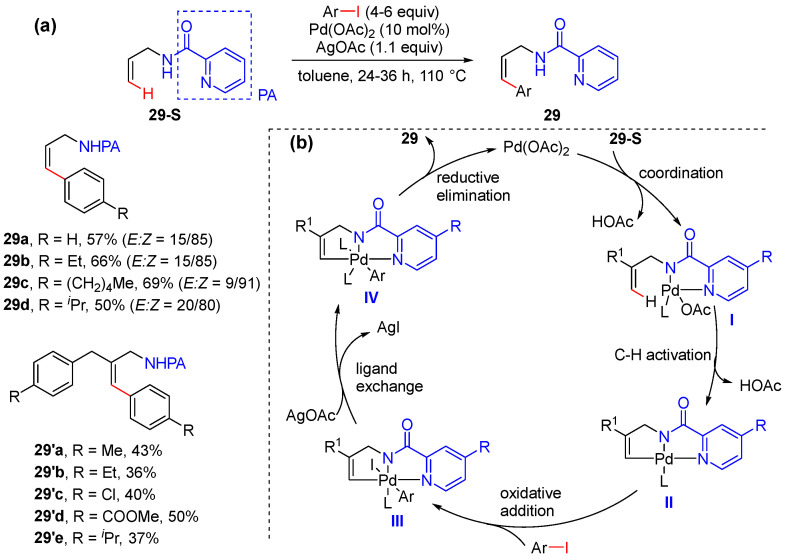
(**a**) Pd-catalyzed alkenyl C–H arylation of allyl amindes. (**b**) Proposed mechanism.

In 2017, Babu’s group reported the picolinamide-assisted olefinic C–H arylation of N-allyl picolinamides, achieved using various aryl iodides under palladium catalysis [50]. This reaction was simply performed in the presence of the catalyst Pd(OAc)_2_ and additive AgOAc in toluene, affording cinnamylamines with moderate to good yields and good to excellent E/Z ratios (Scheme 22a). If the allyl substrate contains both γ-C(sp2)−H and γ-C(sp3)−H bonds, C–H arylation takes place at both γ-C(sp^2^)−H and γ-C(sp3)−H to give bis-arylated cinnamylamines. A Pd(II)−Pd(IV) catalytic cycle was also proposed, as shown in Scheme 22. AgOAc is used to promote the ligand-exchange step to regenerate the catalytically active Pd(II) species (Scheme 22b).

**Scheme 23:**
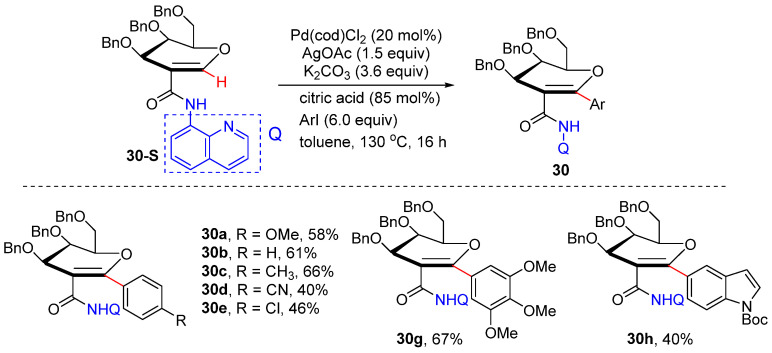
Pd-catalyzed alkenyl C–H arylation of glycosides.

In 2019, Ferry’s group reported a N,N-bidentate-chelation-assisted palladium-catalyzed olefinic C–H arylation of C2-amido glycals onto the anomeric position, affording a novel route to C-aryl/alkenyl glycosides [51]. An aminoquinoline-type directing group was introduced to successfully introduce diverse (hetero)aryl and alkenyl groups at C 1 of the sugar. The protocol is also successfully applied to the synthesis of a dapagliflozin analogue. Alkenyl iodides could also be successfully reacted to afford alkenylation products (Scheme 23).

**Scheme 24:**
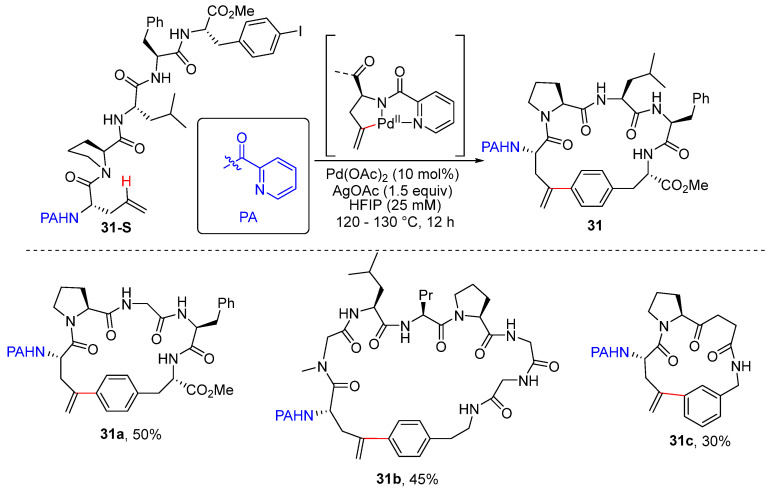
Pd-catalyzed intramolecular alkenyl C–H arylation to prepare cyclophane peptide macrocycles.

Chen and co-workers achieved Pd-catalyzed and picolinamide (PA)-directed intramolecular olefinic C–H arylation using aryl iodides, producing elegant constructions of olefinic and aromatic C-linked cyclophane peptide macrocycles [52]. This protocol is also applicable for intramolecular aromatic C–H arylation. This reaction provides an efficient approach for the preparation of cyclophane-braced structures from easily obtained linear peptide precursors. Notably, the PA group is readily removed by treatment with Zn powder in diluted aqueous HCl (1.5 M) and THF at room temperature to give rise to NH-Fmoc groups (Scheme 24).

### 2.6. Alkenyl C–H Thiolation

Given the important biological and pharmaceutical properties of alkenyl sulfides, there has been a strong impetus to achieve alkenyl C–H thiolation to construct C–S bonds.

**Scheme 25:**
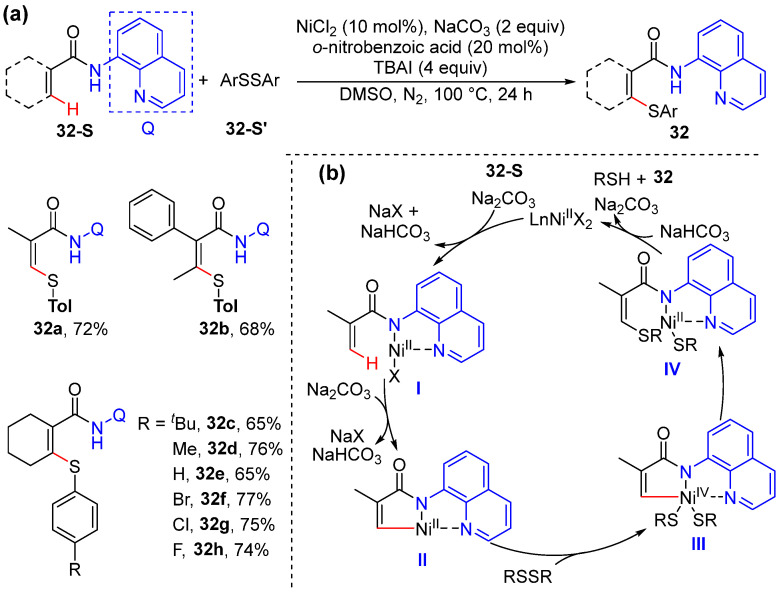
(**a**) Ni-catalyzed alkenyl C–H thiolation of acrylamides. (**b**) Proposed mechanism.

Zhang and co-workers developed a process for nickel-catalyzed aromatic and alkenyl C–H thiolation with diaryl disulfides, using 8-aminoquinoline as the directing group. The protocol exhibits a broad functionality tolerance and substrate scope (Scheme 25a) [53]. The addition of 2,2,6,6-tetramethyl-1-piperidinyloxy (TEMPO) under the standard reaction conditions does not influence the reaction, and this result excludes the possibility of a radical-involved Ni(I)/Ni(III) pathway. A tentative mechanism based on Ni(II)/Ni(IV) cycle was proposed to proceed via an auxiliary 8-aminoquinolyl enabling alkenyl C–H activation to form a five-membered nickelacycle **II,** with the subsequent oxidative addition of disulfide into Ni(II) species **II** and the reductive elimination form the C–S bond. The final ion exchange and protonation produce thiolation product **32** (Scheme 25b).

**Scheme 26:**
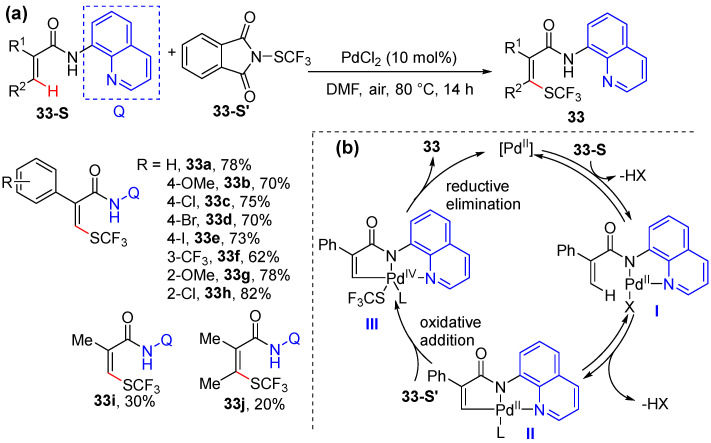
(**a**) Pd-catalyzed alkenyl C–H trifluoromethyl thiolation. (**b**) Proposed mechanism.

Bouillon and Besset achieved the Pd-catalyzed stereoselective trifluoromethyl thiolation of acrylamides using Munavalli reagent **30-S’** as the electrophilic SCF_3_ source [54]. The transformation is also enabled by the chelation assistance of 8-aminoquinoline as the directing group, being simply performed using 10mol% of PdCl_2_ as the catalyst in DMF (Scheme 26a). A broad range of α- and/or β-substituted acrylamides can be smoothly converted to produce specific SCF_3_ substituted alkenes with excellent regio- and stereoselectivity. The reaction is proposed to proceed via the coordination of a bidentate directing group to the Pd catalyst and then C–H cyclometallation via reversible C–H activation to produce **II.** Next, the oxidative addition between **II** and the electrophilic SCF_3_ source occurs to give rise to a putative Pd(IV) species, which undergo reductive elimination to produce **33** and regenerate the catalyst (Scheme 26b).

**Scheme 27:**
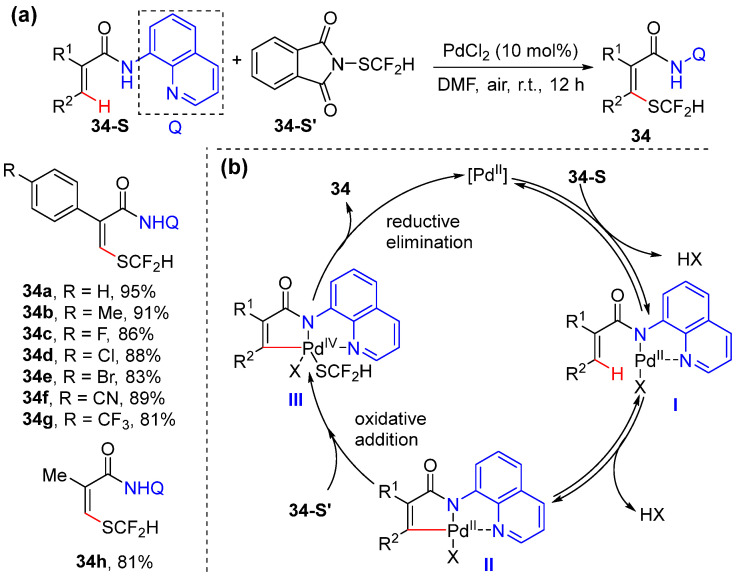
(**a**) Pd-catalyzed alkenyl C–H difluoromethyl thiolation. (**b**) Proposed mechanism.

Pan and He reported the palladium-catalyzed difluoromethyl thiolation of acrylamides, using 8-aminoquinoline as a directing group [55]. The reaction conditions are similar to Bouillon and Besset’s conditions [54]; the reaction is simply performed with 10 mol% PdCl_2_ in DMF at room temperature to access specific SCF_2_H-containing olefin skeletons using Shen’s difluoromethyl thiolation reagent **34-S’** (Scheme 27a). The mechanism includes the coordination of the bidentate directing group to the Pd(II) catalyst; olefinic C–H activation to give palladacycle intermediate **II,** which undergoes oxidative addition with difluoromethyl thiolation reagent to form Pd(IV) species **III**; and the reductive elimination of **III** to yield difluoromethyl thiolation product **34** with the release of the catalyst (Scheme 27b).

### 2.7. Alkenyl C–H Silylation

Vinylsilanes have been widely applied in organic synthesis, material science, and medicines. Olefinic C–H silylation has been used to prepare vinylsilanes.

**Scheme 28:**
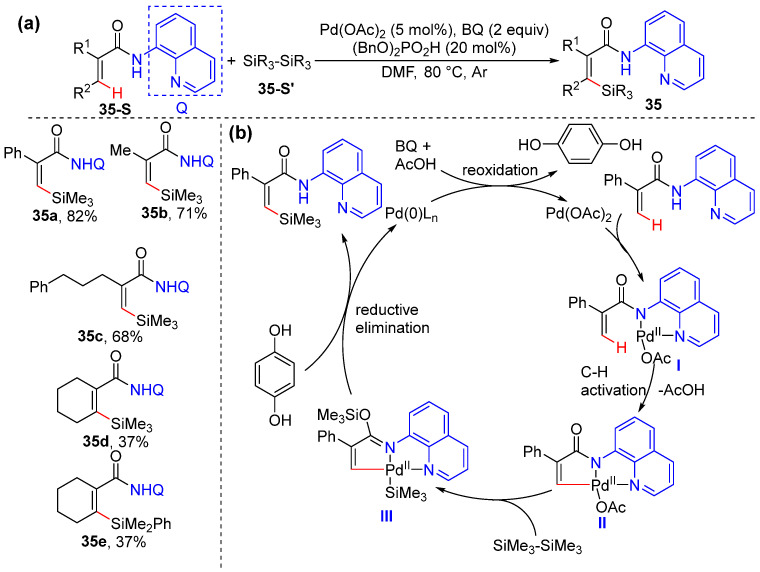
(**a**) Pd-catalyzed alkenyl C–H silylation of acrylamides. (**b**) Proposed mechanism.

Zhang and co-workers reported an efficient method for palladium-catalyzed alkenyl C–H silylation by using disilanes as the silicon source under the assistance of 8-aminoquinoline as the bidentate directing group (Scheme 28a) [56]. This protocol showed good functional group compatibility and efficiently afforded the exclusive synthesis of Z-vinylsilanes, which was confirmed by the isolation of a palladacycle intermediate. The reaction can be successfully applied for gram-scale preparation. The directing group is readily removed to produce a methyl ester, and derivation via ipso-iodination/Suzuki−Miyaura cross-coupling gives rise to trans-stilbene. The reaction is initiated via the chelation of the directing group to the Pd(II) catalyst, followed by ligand exchange, and a C–H concerted metalation−deprotonation (CMD) process takes place to produce a key palladacycle intermediate, **II**. The subsequent insertion of the Si−Si bond and trimethylsilyl group transfer produce intermediate **III**. Reductive elimination followed by a final protonation of the amide give the desired silylation product **35** with the generation of Pd(0) species, which undergo reoxidation to regenerate Pd(II) with the aid of 1,4-benzoquinone (Scheme 28b).

### 2.8. Alkenyl C–H Halogenation

**Scheme 29:**
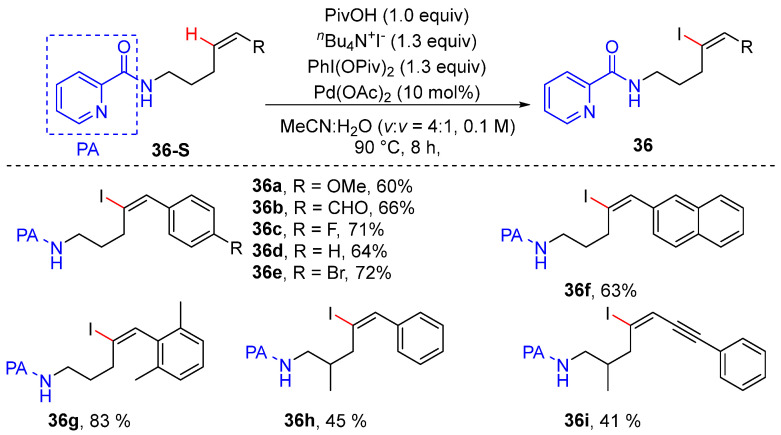
Pd-catalyzed alkenyl C–H iodination of Z-aliphatic alkenes.

In 2019, Carreira’s group achieved the palladium-catalyzed olefinic C–H iodination of aliphatic Z-alkenes using picolinamide as a directing group [57]. The reaction occurred smoothly using 10 mol% palladium acetate, 1.0 equiv. of pivalic acid, and oxidative iodinating agent comprising 1.3 equiv. of tetrabutylammonium iodide and 1.3 equiv. of di(pivaloyloxy) iodobenzene in aqueous acetonitrile. Mechanistic investigations exhibited the formation of a six-membered alkenyl palladacycle intermediate via turnover-limiting olefinic C–H activation, as parallel KIE reactions and intermolecular KIE competition experiments provided *k*_H_/*k*_D_ values of 3.4 and 3.3, respectively (Scheme 29).

### 2.9. Cyclization by Alkenyl C–H Functionalization

Remarkable progress has been made in the preparation of 2-pyridones, benzoisoselenazolones and cyclic imides via Fe-, Ni-, and Co-catalyzed alkenyl C–H functionalization and cyclization [58,59,60].

**Scheme 30:**
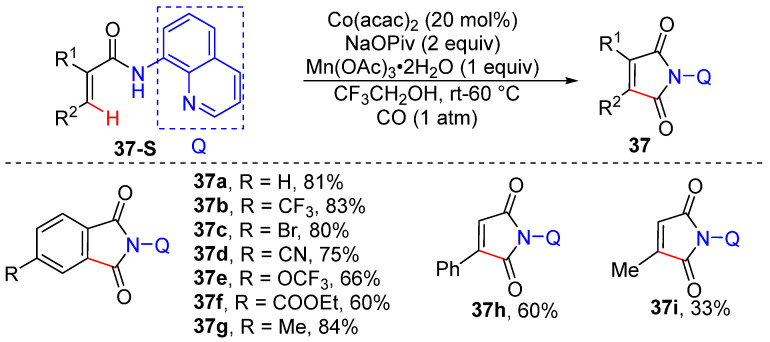
Ni-catalyzed alkenyl C–H carbonylation/cyclization to prepare cyclic imides.

In 2014, Daugulis’s group reported the preparation of cyclic imides from acrylamides via aminoquinoline-directed olefinic C–H carbonylation/cyclization [58]. This reaction is performed with 20 mol% Co(acac)_2_, 2 equivalents of NaOPiv, 1 equivalent of Mn(OAc)_3_•2H_2_O under 1 atm CO in trifluoroethanol. Many functionalities such as CF_3_, CN, CO_2_Et, OCF_3_, and Br are tolerated by this protocol (Scheme 30).

**Scheme 31:**
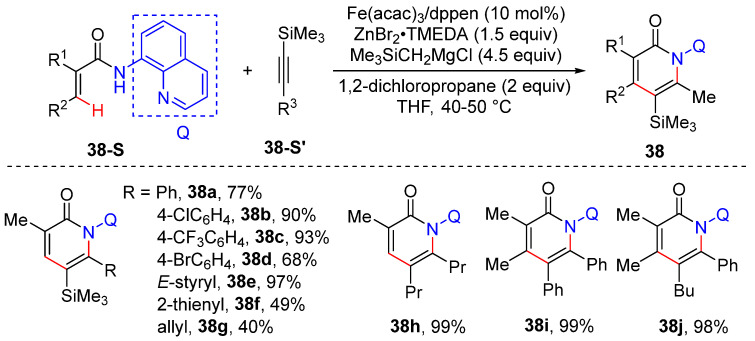
Fe–catalyzed alkenyl C–H activation/cyclization of acrylamides using internal alkynes.

In 2017, Nakamura and Ilies developed a method of iron-catalyzed C–H bond cleavage and C−N bond formation to yield 2-pyridone **38** and isoquinolones from arylamides and internal alkynes [59]. Unsymmetrical alkynes also produce the pyridone derivative with high regioselectivity, presumably due to the sensitivity of the iron catalyst to the reaction’s steric effects. The transformation is performed using Fe(acac)_3_ as a catalyst, cis-1,2-bis(diphenylphosphino) ethylene (dppen) as a ligand, bis(trimethylsilylmethyl)zinc as a base, and 1,2-dichloropropane as an oxidant. The reaction shows no formation of byproducts such as the coupling of acrylamides and silylmethyl anion or the homocoupling of the silylmethyl reagent (Scheme 31).

**Scheme 32:**
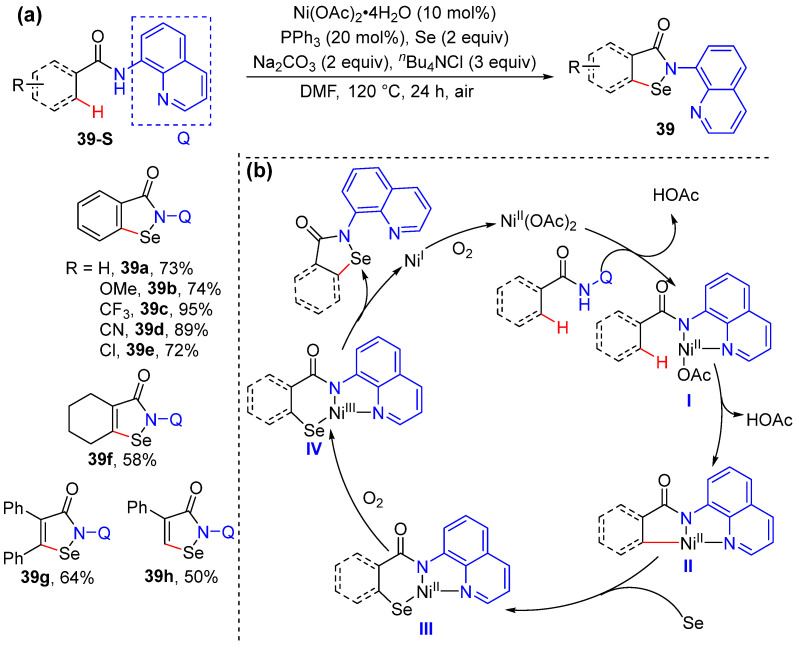
(**a**) Ni-catalyzed alkenyl C–H selenation/cyclization of acrylamides. (**b**) Proposed mechanism.

Nishihara and coworkers developed a method for the Ni-catalyzed direct selenation/cyclization of acrylamides and benzamides bearing an auxiliary 8-quinolyl using elemental selenium to produce benzoisoselenazolone **39** under aerobic conditions (Scheme 32a) [60]. The reaction proceeds in the presence of Ni(OAc)_2_· 4H_2_O and PPh_3_ as the catalyst, Na_2_CO_3_, and Bu_4_NCl in DMF under atmospheric air. The addition of Bu_4_NCl is required presumably due to its aiding in dissolving the elemental selenium in organic solvents. A possible mechanism is proposed to be initiated by a base-promoted ligand exchange between the nickel (II) catalyst and the substrate, which then undergoes C–H activation to produce a nickelacycle intermediate. The insertion of elemental selenium into a carbon−nickel bond forms intermediate **III**, which undergoes aerobic oxidation to produce cyclic nickel (III) species **IV**. A final reductive elimination delivers the nickel (I) complex and the product **39**. The nickel (I) complex is converted to nickel (II) species by oxidation in air (Scheme 32b).

### 2.10. N,O-Bidentate-Chelation-Assisted Alkenyl C–H Functionalization

**Scheme 33:**
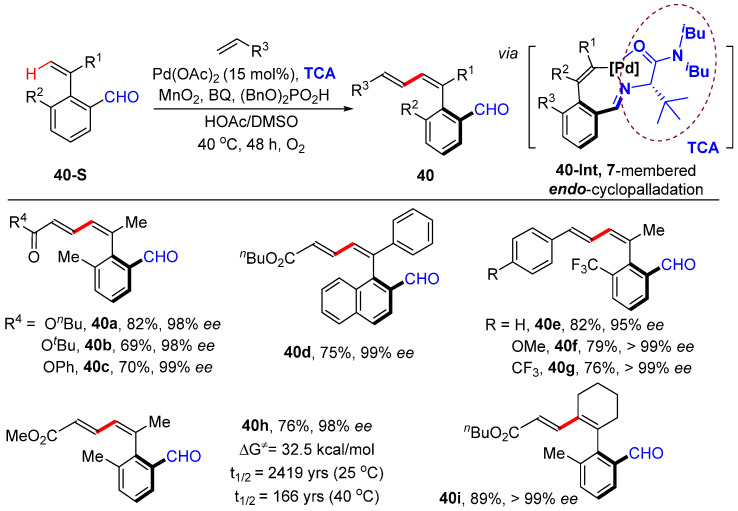
Pd-catalyzed asymmetric β-C–H alkenylation of aryl alkenes.

Zhong and Zhang achieved the Pd-catalyzed atroposelective β-olefinic C–H alkenylation of aryl alkene under N,O-bidentate chelation assistance [61]. This protocol introduces aldehyde- and amino-acid-derived transient chiral auxiliaries, which is proceeded by seven-membered endo-cyclometallation **40-Int**, producing axially chiral aryl 1,3-dienes bearing aldehyde in up to 99% yields and with excellent enantioselectivities (up to >99% ee). The products are oxidized to produce axially chiral carboxylic acid without erosion of enantioselectivity, which were successfully used as chiral ligands in Co(III)-catalyzed asymmetric 1,4-addition reactions (Scheme 33).

**Scheme 34:**
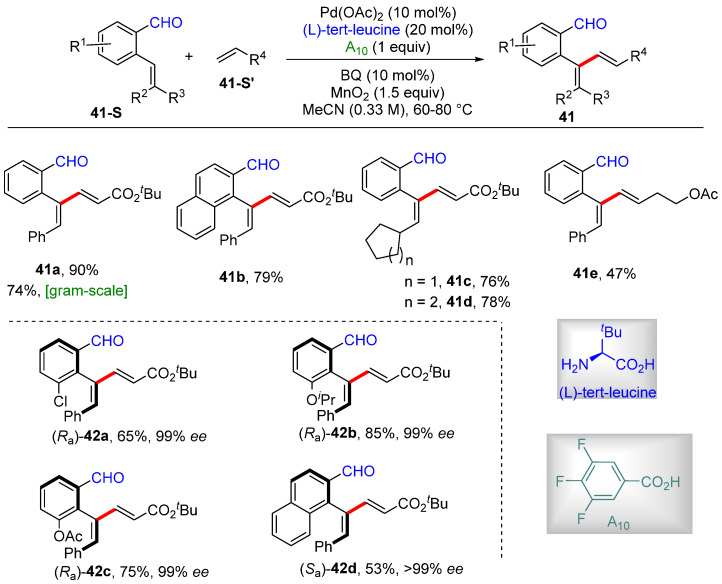
Pd–catalyzed (asymmetric) α-C–H alkenylation of aryl alkenes according to Engle.

Engle’s group reported the Pd-catalyzed α-C–H alkenylation of styrenes using a N,O-bidentate-chelation-assisted transient directing group derived from aldehyde and amino acid, producing aryl 1,3-diene with excellent site- and E/Z-selectivity [62]. 3,4,5-Trifluoro benzoic acid was used as the CMD promoter and tert-leucine as the transient directing group. Also, the enantioselective α-C–H alkenylation of styrenes was realized by introducing chiral L-tert-leucine as a transient directing group (Scheme 34).

**Scheme 35:**
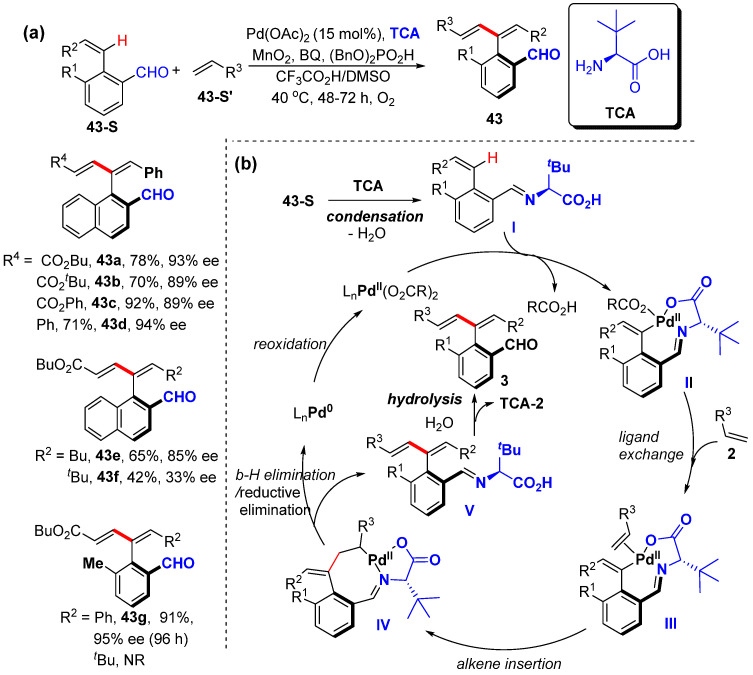
(**a**) Pd–catalyzed asymmetric α-C–H alkenylation of styrenes. (**b**) Proposed mechanism.

After advances in the asymmetric β-C–H functionalization of styrenes [61], Zhong and Zhang also reported the asymmetric α-C–H alkenylation of aryl alkenes to prepare axially chiral aryl 1,3-diene (Scheme 35a) [63]. This protocol is also assisted by an aldehyde/L-t-leucine-derived transient chiral auxiliary, which is performed using Pd(OAc)_2_ as the catalyst, amino acid as the transient chiral auxiliary (**TCA**), 1.5 equivalents of MnO_2_ and BQ as the oxidants, and (BnO)_2_PO_2_H as an additive in mixed HOAc/DMSO under an O_2_ atmosphere, affording excellent enantioselectivity. Also, axially chiral aryl carboxylic acids are obtained from axially chiral aryl dienes via simple oxidation with NaClO_2_/NaH_2_PO_4_, which are used in the asymmetric 1,4-addition of indole with maleimide under Co(III) catalysis. A plausible mechanism is proposed to start with condensation between aldehyde and amino acid **TCA** to afford imine **I**, which coordinates with palladium species and undergoes C–H activation to generate a six-membered exo-palladacycle **II**. Ligand exchange and alkene insertion produce eight-membered exo-palladacycle **IV**. β-H elimination and imine hydrolysis produce axially chiral styrene **43** and regenerate amino acid **TCA**. The reductive elimination and reoxidation of the Pd^0^ species regenerate active Pd(II) species to enter the next catalytic cycle (Scheme 35b).

## 3. Conclusions

N,N- and N,O-bidentate-chelation-assisted C–H functionalization by cyclometallation event has been rapidly increasing applied in the past decade. This review focused on alkenyl C–H functionalization to produce valuable olefinic products with excellent site- and stereoselectivity via endo-cyclometallation and exo-cyclometallation processes. The bidentate-chelation-assistance strategy is a robust synthesis protocol that is complementary to monodentate-chelation-assisted olefinic C–H functionalization. Despite remarkable progress, most of the developed protocols require catalysis with expensive transition metals such as palladium and the tedious installation/removal of the bidentate directing group, which greatly hinder their practical usage, so the development of transient directing group-promoted (multiple) alkenyl C–H functionalization using catalysis with inexpensive transition metals such as Co and Ni is still highly desirable. In-depth investigations on the mechanisms and late-stage C–H functionalization of biologically active molecules are ongoing in this exciting and developing area.

## Data Availability

No new data were created.
